# Targeted Manipulation of Serotonergic Neurotransmission Affects the Escalation of Aggression in Adult Male *Drosophila melanogaster*

**DOI:** 10.1371/journal.pone.0010806

**Published:** 2010-05-24

**Authors:** Olga V. Alekseyenko, Carol Lee, Edward A. Kravitz

**Affiliations:** 1 Neurobiology Department, Harvard Medical School, Boston, Massachusetts, United States of America; 2 Albert Einstein College of Medicine, New York City, New York, United States of America; Columbia University, United States of America

## Abstract

Dopamine (DA) and serotonin (5HT) are reported to serve important roles in aggression in a wide variety of animals. Previous investigations of 5HT function in adult *Drosophila* behavior have relied on pharmacological manipulations, or on combinations of genetic tools that simultaneously target both DA and 5HT neurons. Here, we generated a transgenic line that allows selective, direct manipulation of serotonergic neurons and asked whether DA and 5HT have separable effects on aggression. Quantitative morphological examination demonstrated that our newly generated *tryptophan hydroxylase (TRH)-Gal4* driver line was highly selective for 5HT-containing neurons. This line was used in conjunction with already available Gal4 driver lines that target DA or both DA and 5HT neurons to acutely alter the function of aminergic systems. First, we showed that acute impairment of DA and 5HT neurotransmission using expression of a temperature sensitive form of dynamin completely abolished mid- and high-level aggression. These flies did not escalate fights beyond brief low-intensity interactions and therefore did not yield dominance relationships. We showed next that manipulation of either 5HT or DA neurotransmission failed to duplicate this phenotype. Selective disruption of 5HT neurotransmission yielded flies that fought, but with reduced ability to escalate fights, leading to fewer dominance relationships. Acute activation of 5HT neurons using temperature sensitive dTrpA1 channel expression, in contrast, resulted in flies that escalated fights faster and that fought at higher intensities. Finally, acute disruption of DA neurotransmission produced hyperactive flies that moved faster than controls, and rarely engaged in any social interactions. By separately manipulating 5HT- and DA- neuron systems, we collected evidence demonstrating a direct role for 5HT in the escalation of aggression in *Drosophila*.

## Introduction

Aggression in competition for resources is seen in essentially all species of animals. In our fruit fly model of aggression [1]–[3] pairs of males form and maintain stable hierarchical relationships. Dopamine (DA) and serotonin (5HT) have been implicated in the regulation of aggression in a wide variety of animal models including humans [4]–[8]. The fruit fly system offers the advantage of having powerful genetic methods already available that can be used to unravel the roles of amine neurons in behavior.

The rate-limiting enzymes in the biosynthesis of the amines in *Drosophila* are tyrosine hydroxylase (TH) for DA [9], [10] and tryptophan hydroxylase (dTPH and dTRH) for 5HT [11]–[13]. The final common step in the biosynthesis of both amines involves the enzyme dopa decarboxylase (DDC, [14], [15]). Flies deficient for the gene DDC [16]–[18] and DA null mutants [9], [19] die during development. The use of the binary *Gal4/UAS* system [20] allows chronic or acute manipulation of the function of amine neurons at different times during development and in adults. Both *DDC-Gal4* [21] and *TH-Gal4* [22] transgenic lines have been generated for this purpose. A line that drives expression in serotonergic neurons has been mentioned before [23] but not characterized. To date, putative roles for 5HT neurons have been suggested in sleep [24], aggression [25] and memory [26] using pharmacology and genetic tools containing DDC and TH regulatory elements.

In these studies, we generated and characterized a new *TRH-Gal4* transgenic line that drives expression of effector genes in 5HT-containing neurons. The line is more selective and more complete in its overlap with neuronal 5HT-immunostaining than the previous TPH driver ([23], see Results and Supplementary Information), thereby allowing specific and efficient manipulation of the 5HT-positive population of neurons. Using the *TRH-, TH-* and *DDC-Gal4* lines in crosses with a temperature sensitive dominant negative *dynamin line* (*UAS-shi^ts1^*, [27]), we asked whether effects on aggression of DA and 5HT neuron functions were separable from each other. With the *Ddc-Gal4* driver, acute functional impairment of DA- and 5HT-neurotransmission produced flies that engaged exclusively in low-intensity interactions without escalating to higher intensity levels, and therefore, that never led to the establishment of dominance relationships. Selective disruption of DA neuron function yielded hyperactive flies that rarely initiated any social behavior. Selective blockade of 5HT neurotransmission did affect aggression, yielding flies with a reduced ability to escalate to higher intensity levels. In this case, therefore, significantly fewer dominance relationships were established. To confirm the importance of 5HT neurotransmission in the proper escalation of fights we activated 5HT neurons through expression of temperature-sensitive dTrpA1 channels [28], and observed more intense fights with shorter escalation time compared to controls.

## Results

### Simultaneous disruption of dopaminergic and serotonergic neuron function abolishes higher-intensity aggression

The enzyme dopa decarboxylase (DDC) is expressed in both serotonergic and dopaminergic neurons in the fruit fly nervous system. Although we focus on the two amines, DDC also has been reported to be expressed in neurons containing corazonin and possibly other unknown substances [26], [29], [30]. By using a *DDC-Gal4* driver line in crosses with *UAS-shi^ts1^* flies, we attempted to acutely alter the function of neurons expressing both amines in adult male flies. Progeny of these crosses (experimental flies) should have normal dynamin function (coded for by *shibire*) in 5HT- and DA-containing neurons at the permissive temperature (19°C); at the restrictive temperature (30°C), synaptic vesicle recycling should be blocked causing a depletion of synaptic vesicles and an associated reduction in transmitter release [27], [31]. Using our standard fight chambers [1] and a headless female, light and food as resources, we paired size matched 5–7 day old males and scored fights at both restrictive and permissive temperatures. The experimental flies at the permissive temperature served as “temperature controls”. As a second set of controls (“genetic controls”, see Methods), fights between pairs of flies carrying the *UAS-shi^ts1^* but not the GAL4 driver transgene were carried out at both temperatures.

**Figure 1:**
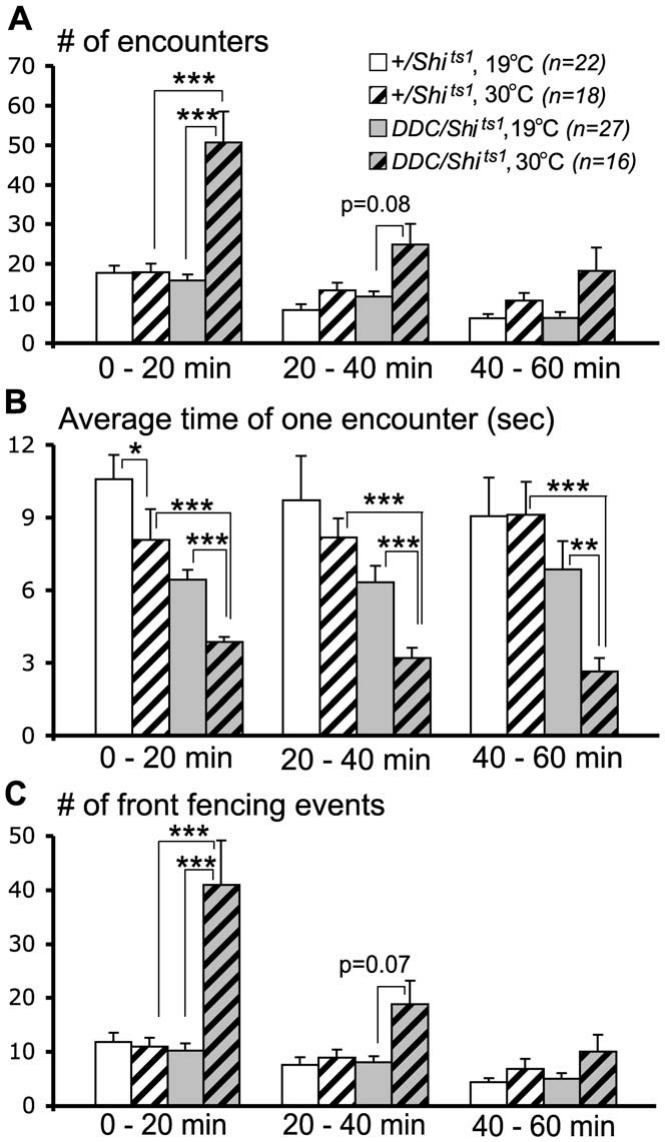
Simultaneous disruption of serotonergic and dopaminergic neurotransmission results in increased numbers of low intensity encounters. (A) The numbers of encounters were significantly increased in *DDC-Gal4/UAS-sh^ts1^* flies at the restrictive temperature (gray striped bar) during the first 20 min of the fight compared to the same genotype of flies at the permissive temperature (gray bar), or genetic controls at the restrictive temperature (striped bar). Effects were less pronounced and not statistically significant during the remaining 40 min of a fight. (B) The average time of each encounter was shortened in *DDC-Gal4/UAS-shi^ts1^* flies at the restrictive temperature (gray striped bars) for the entire fight. (C) The encounters between pairs of *DDC-Gal4/UAS-shi^ts1^* males at the restrictive temperature consisted mainly of fencing behavior. As above, results were significant only for the first 20 min.*p<0.05; **p<0.01; ***p<0.001, analyzed by nonparametric two-independent-sample Mann-Whitney U-test.

The results showed that the numbers of encounters between experimental flies more than doubled at the restrictive temperature while the average duration of each encounter was greatly shortened throughout the duration of a fight when compared to fights between flies of the same-genotype at the permissive temperature (Fig 1, A–B). Effects of this type were not seen in the “genetic controls” at elevated temperatures, except that the average time of single encounters was lowered during the first 20 min of fights between genetic control flies at 30°C. Low-intensity front fencing events more than tripled in number in the experimental flies at 30°C (Fig 1, C). The differences were most dramatic during the first 20 min of a fight when flies normally show the highest numbers of encounters [3]. Despite increases in the number of interactions between the flies, fights did not escalate to mid-intensity levels (no lunges or holding were observed) in the experimental group at the higher temperature. Escalation was seen in all control pairings under all experimental conditions, which was accompanied by the anticipated establishment of hierarchical relationships (Table 1). Since the flies with diminished 5HT- and DA-neuron function did not lunge, no hierarchical relationships resulted from these pairings (Table 1).

**Table 1:** Disruption of aminergic neurotransmission affects lunge and dominance rates.

	*w^1118^;Shi^ts1^/+*	*DDC/Shi^ts1^*	*w^1118^(CS);Shi^ts1^/+*	*TRH/Shi^ts1^*
***Lunging, 19°C***	86%	63%	87%	78%
***Lunging, 30°C***	83%	0%***	62%	29%**
***Dominance, 19°C***	63%	48%	1)31%	67%
***Dominance, 30°C***	50%	0%***	1)33%	10%***

The data in the table are presented as the percentage of fights in each category during which lunging behavior and the establishment of dominance relationship were seen. Pairs of *DDC-Gal4/UAS-shi^ts1^* males at the restrictive temperature (*DDC/Shi^ts1^*, 30°C) did not lunge or establish dominance relationships. Among *TRH-Gal4/UAS-shi^ts1^* males at the restrictive temperature (*TRH/Shi^ts1^*, 30°C), the percentages of pairs that displayed lunging behavior and formed dominance relationship were significantly reduced.

1) Low dominance rates were observed in *w^1118^(CS);Shi^ts1^/+* genetic controls. At both high and low temperature conditions this might be due to a reduction in the escalation of fights of this genotype to mid-intensity levels (reduced number of lunges). Normal dominance rates were observed when fights between genetic control groups were carried out at 25°C (see Table S1).

**p<0.01;

***p<0.001 vs. same genotype at the permissive temperature (19°C), analyzed by nonparametric two-independent-sample Mann-Whitney U-test.

**Figure 2:**
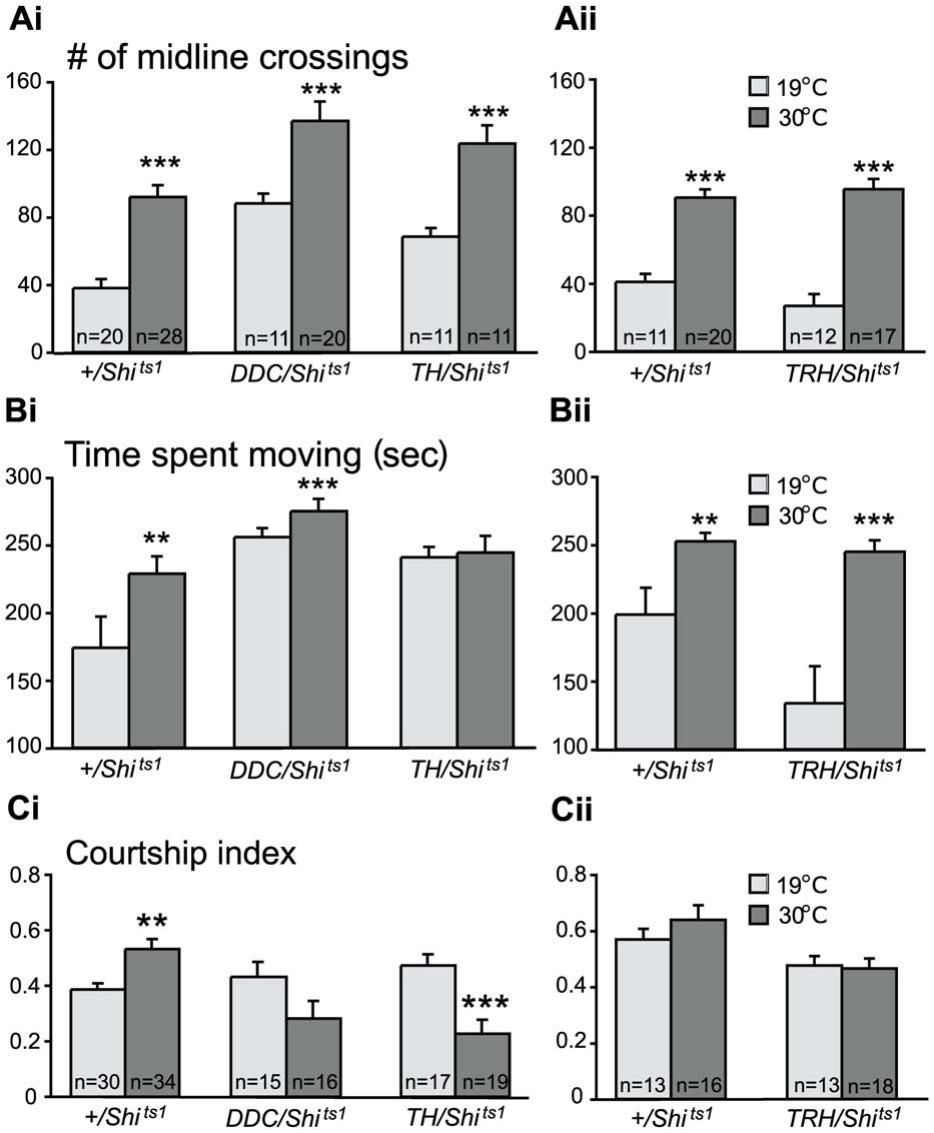
Effects of disrupted amine neuron function on locomotion and courtship behavior. (Ai,Aii) Flies of all genotypes examined showed parallel temperature-induced increases in the numbers of midline crossings in a locomotion assay. (Bi,Bii) A temperature shift from 19°C to 30°C increased the time that flies spent moving during the locomotion assay in all groups except in males with disrupted dopaminergic neurotransmission (*TH/Shi^ts1^*, 30°C). (Ci) In courtship assays, genetic controls (*+/Shi^ts1^*, 30°C) showed a significant increase in courtship index at the elevated temperature. Flies with lowered function of both amine systems (*DDC/Shi^ts1^*, 30°C) had a slight decrease rather than an increase in CI as seen in controls. With selective interference with dopaminergic function (*TH/Shi^ts1^*, 30°C), however, male flies showed a significant reduction in courtship index. (Cii) By contrast, disruption of serotonergic neurotransmission had no effect on the courtship index. **p<0.01; . ***p<0.001 vs. same genotype at the permissive temperature (19°C), analyzed by nonparametric two-independent-sample Mann-Whitney U-test.

Next, other aspects of behavior, including locomotion, courtship, geotaxis and phototaxis, were examined in all experimental pairings at high and low temperatures. No significant differences were found in geotaxis or phototaxis behavior between “genetic controls” and any of the experimental genotypes: therefore, data for these tests are not included here or in later experiments. Elevated temperatures did lead to significant increases in locomotion both in the numbers of midline crossings and in the time spent moving in assay chambers, but these temperature effects were seen in the genetic controls as well as in experimental groups (Fig 2, Ai, Bi). This is not surprising since temperature has profound effects on behavioral performance in *D. melanogaster* [32]. Genetic controls also showed a significant increase in the courtship index at elevated temperatures (Fig 2, Ci), but if anything, flies with lowered DA- and 5HT-neuron function showed a trend towards a decrease in the courtship index at the restrictive temperature (the value did not reach significance in this set of experiments).

Thus, the following effects resulted from disrupting neurotransmission in 5HT and DA containing neurons using the *DDC-Gal4* driver: (a) a failure of fights to escalate to higher intensity levels; (b) a failure to establish hierarchical relationships; (c) an increased number of short low-intensity encounters; and a small decrease, rather than an increase in C.I. seen at an elevated temperature in control flies. The question remained whether these effects were due to both amines. If so, would it be possible to distinguish the separate contributions of 5HT and DA neuron systems to the observed phenotype?

### Generation of *TRH-Gal4* lines that selectively target 5HT neurons

Previous investigators estimated the contributions of 5HT neurons to behavior in *D. melanogaster* either by pharmacological means, or by comparing the behavioral effects of *DDC-* and *Tyrosine hydroxylase* (*TH)-Gal4* driven expression of effector genes [24], [25]. This presupposes that the complex actions of two multifunctional neurohormonal systems are additive; however, no compelling evidence exists supporting that contention. Therefore, we sought to examine the behavioral consequences of manipulating each neurohormonal system separately and then comparing the results obtained with data gathered when the two systems were manipulated together.

**Figure 3:**
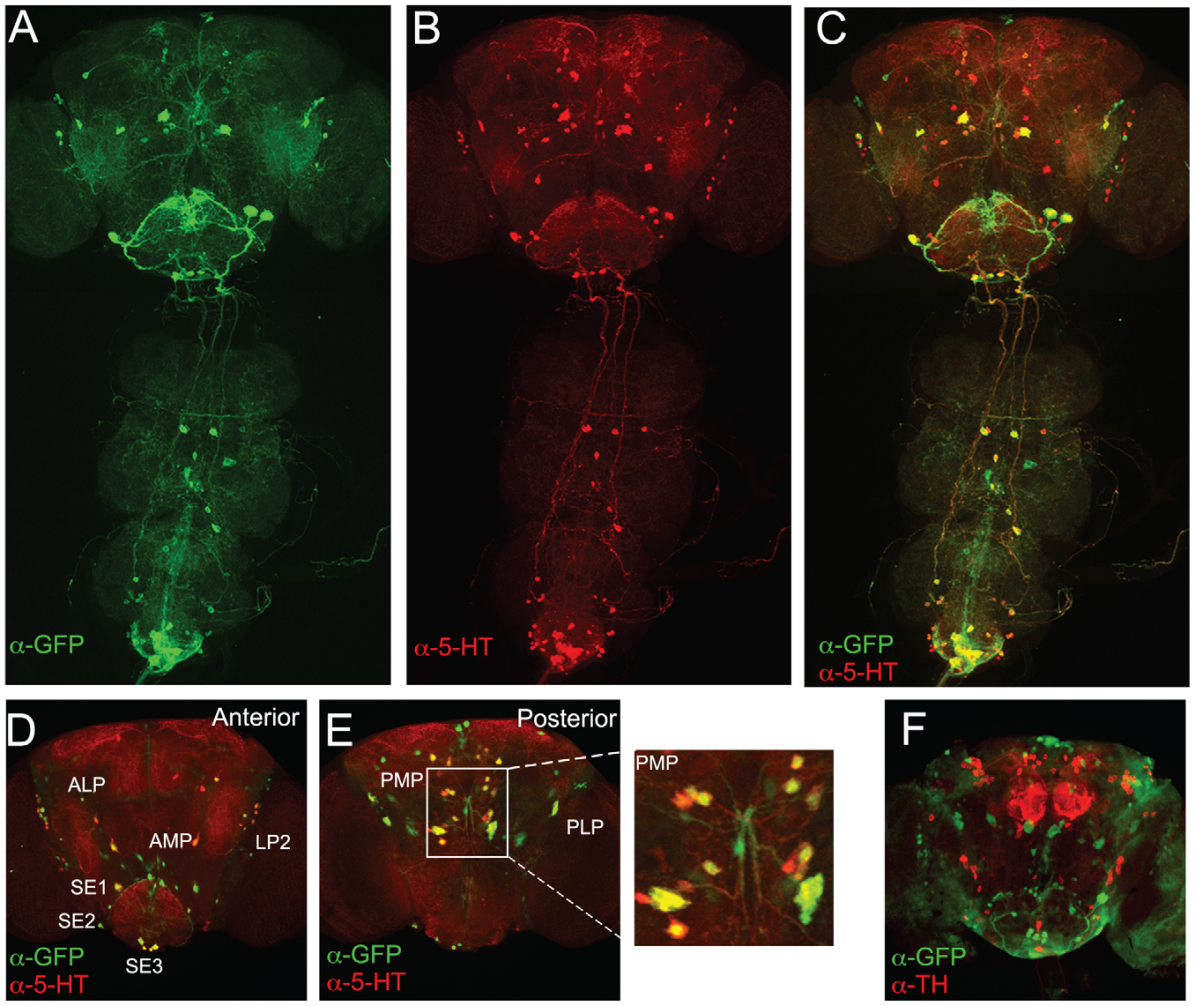
A comparison of *TRH-Gal4* driven *GFP* expression and 5HT immunostaining in male *Drosophila* brains. (A–C) *TRH-Gal4* (3^rd^ chromosome line) driven *mCD8∶GFP* signal (A), 5HT immunostaining (B) and overlay (C) of the staining patterns in the brain and the ventral cord of an adult male. (D–F) Anterior (D) and posterior (E) adult male brain 5HT clusters visualized by *TRH-Gal4* driven nuclear *nls∶*GFP (green) expression and 5-HT immunostaining (red). Note that anterior AMP cells are visible with *UAS-nls∶GFP* (D), but not with *UAS-mCD8∶GFP* (A). (F) The absence of overlap between *TRH-Gal4* driven *UAS-nls∶GFP* (green) and DA-containing neurons visualized by TH immunostaining (red).

Towards this goal, we cloned short and long regulatory sequences of the *Trh* gene that encodes the nervous system specific *tryptophan hydroxylase* enzyme (CG9122, [12], [13]) upstream of a *Gal4* coding region. Twelve viable *TRH-Gal4* fly lines were generated, and *Gal4* expression patterns were visualized for each line by crossing these flies to *UAS-nls∶GFP* (for nuclear labeling) or *UAS-mCD8∶GFP* (for membrane labeling). The GFP fluorescence in progeny was examined in brains and in ventral nerve cords (VNC). Ten lines derived from the short regulatory sequence showed variable and partial overlap between *TRH-Gal4* driven GFP expression and immunostaining for 5HT (Fig. S1, D–I). Two lines, derived from the long regulatory sequence of *Trh*, had more specific patterns of GFP expression that almost completely overlapped with 5HT immunostaining (Fig. 3, A–C for the line on 3^rd^ chromosome; Fig. S1, A–C for the line on 2^nd^ chromosome). For the *TRH-Gal4* line on the 3^rd^ chromosome, nuclear GFP labeling was seen in all of the previously reported adult brain 5HT clusters (Fig. 3, D–E): **LP2**, **SE1**, **SE2** and **SE3** anterior clusters [17]; the more recently classified **AMP** and **ALP** anterior clusters [26]; and groups of cells forming the **PMP** and **PLP** posterior clusters [26]. Co-labeling between GFP and 5HT immunostaining was present in 75–100% of the neurons forming each 5HT cluster. See Table S2 for numbers of labeled serotonergic neurons in each cluster in comparison with previously used genetic approaches. Also see Fig. S2 for close-up views of 5HT clusters visualized by *TRH-Gal4* and *TPH-Gal4* [23]. We did not observe co-localization between GFP-expressing cells and tyrosine hydroxylase antibody staining, indicating that the new line does not drive expression in dopaminergic neurons (Fig 3, F). This *TRH-Gal4* line, mapped to the third chromosome, was used in the behavioral experiments that follow.

### Selective disruption of serotonergic neurotransmission reduces the ability to escalate fights

**Figure 4:**
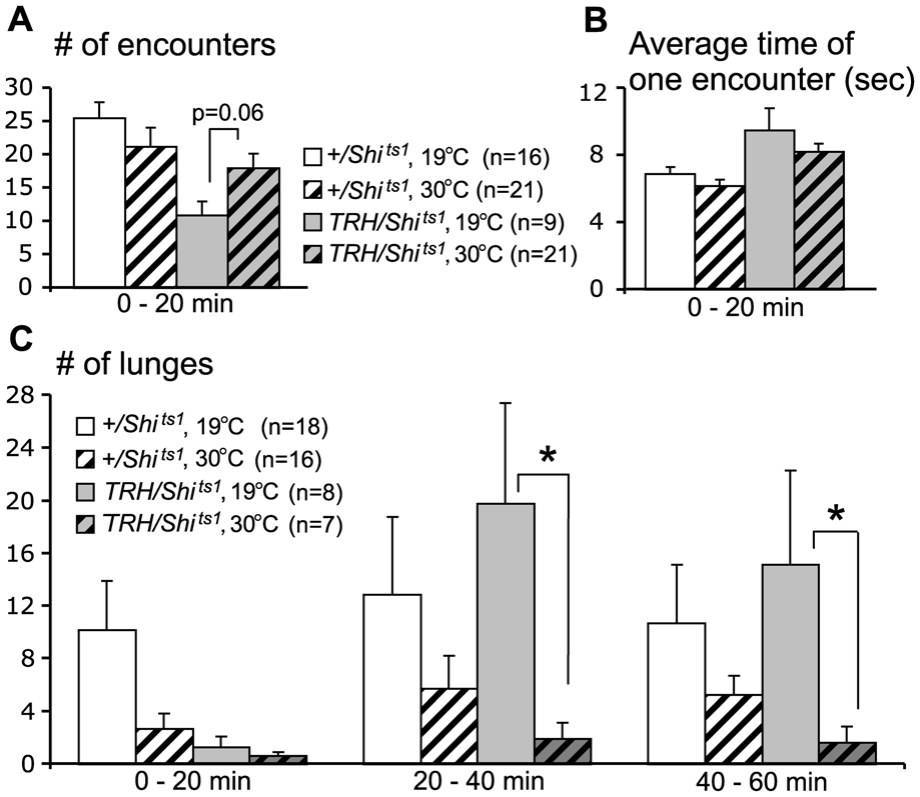
Selective disruption of serotonergic synaptic transmission results in less aggressive flies. No significant differences were seen in the numbers of encounters (A) or in the average duration of each encounter (B) throughout a fight (shown for the first 20 min only). (C) *TRH-Gal4/UAS-shi^ts1^* flies at the restrictive temperature (gray striped bars), however, did show decreased numbers of lunges compared to the experimental males at the permissive temperature (gray bars). This effect was most pronounced at the 20–40 and 40–60 min time windows. Decreases also were seen in the numbers of lunges in genetic control flies, but these were not significant, and were related to switching flies to an elevated temperature. *p<0.05 vs. same genotype at the permissive temperature (19°C), analyzed by nonparametric two-independent-sample Mann-Whitney U-test.

In order to reduce 5HT release from serotonergic neurons alone, we drove the expression of *UAS-shibire^ts1^* (*UAS-shi^ts1^*) using the *TRH-Gal4* line. In contrast to the results obtained with the *DDC-Gal4* driven progeny, the numbers of encounters and the average duration of each encounter did not differ significantly from either temperature or genetic controls during any of the three 20 min time periods (Fig. 4, A, B, data shown for the first 20 min period). Experimental flies did show an increase in the numbers of low-intensity fencing events during the first 20 min of a fight compared to the same genotype at the permissive temperature (7.3±2.0 at 19°C vs. 16.7±2.6 at 30°C; Mann-Whitney U = 50.5, p = 0.046). The most important difference in comparing *DDC-Gal4* and *TRH-Gal4* datasets was that we observed some escalation to higher intensity levels of aggression in fights between flies with disrupted 5HT neurotransmission.

In genetic control fights, the lunge rate and numbers of lunges appeared to be slightly reduced at 30°C compared to 19°C, but they were not statistically different from each other (Table 1; Fig. 4, C). The experimental flies at 19°C showed similar numbers of lunges as the genetic controls at that temperature. At 30°C, experimental flies had large and significant reductions in lunge rate and numbers (Table 1; Fig. 4, C). Another indicator of mid-intensity aggression, number of “holds”, dropped from 0.88±0.4 at the permissive temperature to zero at the restrictive temperature (Mann-Whitney U = 14.0, p = 0.037). Thus, at the restrictive temperature, fights between pairs of experimental flies could escalate to mid-intensity levels, but the frequency of usage of these behavioral patterns was dramatically reduced. As a consequence, hierarchical relationships, which require lunging behavior, were established in a smaller percentage of the fights (67% at the permissive vs. 10% at the restrictive temperature, Table 1).

In examining other aspects of behavior, both genetic control and experimental flies showed anticipated temperature-induced increases in locomotion (Fig 2, Aii, Bii), but no changes in courtship behavior were found at the elevated temperature (Fig 2, Cii). Males with reduced 5HT neurotransmission also showed an unusual pattern of movement that was not seen in control flies–when moving, these flies suddenly collapsed, rolled to the side, then sprung up and continued what they were doing (Movie S1). To confirm that the observed behavioral phenotype was not due to P-element positional effects, we built a line carrying two *TRH-Gal4* transgenes derived from the long regulatory sequence of *Trh* gene. With this “double” *TRH-Gal4* line we quantified the “movement” phenotype and observed an increase in the numbers of flies showing the phenotype from 22 to 50% (data not shown). When genetic control males were paired with experimental flies at the restrictive temperature, the experimental flies consistently showed the “movement” phenotype and reduced numbers of lunges. Despite these defects, experimental flies did initiate lunging behavior in 60% of the fights (data not shown) suggesting that their “willingness to fight” was not compromised by the motor dysfunction phenotype.

### Selective activation of serotonergic neurons increases aggressiveness of flies

**Figure 5:**
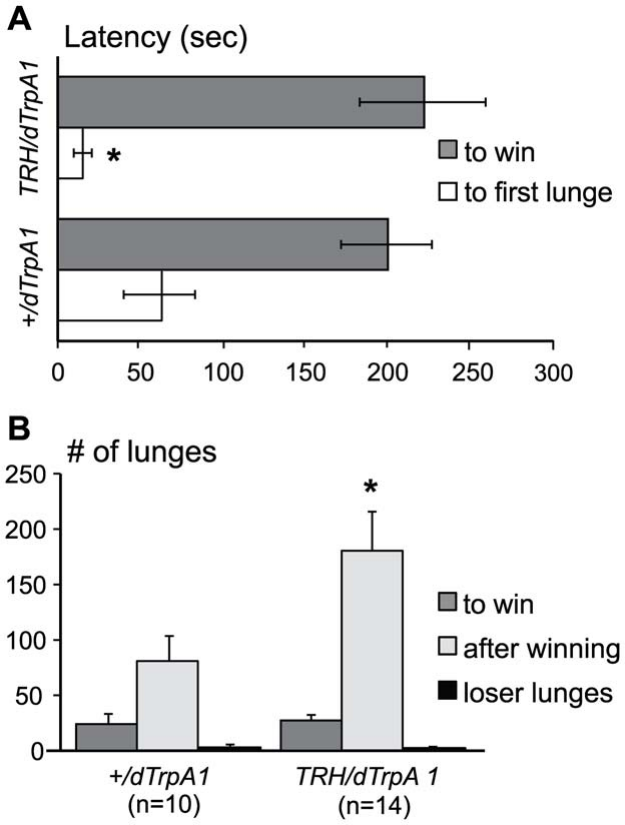
Selective activation of serotonergic neurons increases the intensity of aggression. *TRH-Gal4/UAS-dTrpA1* flies after 15 min at 26°C had a significantly shorter latency to the first lunge (A), and kept lunging more then controls after dominance status was established (B). *p<0.05 vs. genetic control group, analyzed by nonparametric two-independent-sample Mann-Whitney U-test.

To enhance rather than reduce the function of serotonergic neurons, we expressed the dTRPA1 channel in serotonergic neurons by crossing *TRH-Gal4* flies with the *UAS-dTrpA1* line [28]. dTrpA1 channels are TRP family cation channels that open at specific threshold temperatures: these can be used to selectively activate neurons in living animals by small temperature increases [28]. The dTrpA1 channel opens at +26°C [33], allowing manipulation of neuronal activity in a normal physiological temperature range, thereby avoiding the high temperatures required with the temperature sensitive *shibire* gene product. All pairs of flies in which serotonergic neurons were activated in this way displayed mid-intensity aggression, and 90% of the pairings resulted in the establishment of dominance relationships. These results were not statistically different from control fights between pairs of flies carrying the *UAS-dTrpA1* transgene alone. The latency to establish dominance, the numbers of lunges before winning and the low numbers of lunges displayed by ”loser” flies were not different from control fights (Fig. 5, A–B), suggesting that the ability to form and maintain hierarchical relationships was not compromised by acute activation of serotonergic circuits. However, the latency to the first lunge (Fig. 5, A) was significantly shortened and the numbers of lunges performed by “winners” after establishing dominance were dramatically increased (Fig. 5, B) in experimental pairings, suggesting that flies escalate aggression faster and fight at higher intensity levels when serotonergic neurons are activated. Similar results obtained with another available *UAS-dTrpA1* line (on 2nd chromosome, data not shown) suggest that the reported phenotype comes from the manipulation of 5HT-neuron function rather than from positional effects of the *UAS-dTrpA1* transgenes.

### Selective disruption of dopaminergic neurotransmission produces hyperactive flies

Finally, to selectively alter the function of DA neurons, we used a well-established *TH-Gal4* line [22] and the *UAS-shi^ts1^* genetic tool. Although the experimental flies fought normally at the permissive temperature, surprisingly, they did not land on the food cup at the restrictive temperature. Instead, they were in almost constant motion while in the fighting chamber. To ask whether these flies would fight if we forced them to interact more, fights were set up in smaller chambers. Under these conditions, flies with disrupted DA neurotransmission landed on food cups, but their levels of social interactions remained low compared to genetic and temperature control flies (data not shown).

Chronic silencing of DA neurons in flies using tetanus toxin to prevent transmitter release reportedly causes a dramatic hyperexcitablility [22]. In our locomotion assay, acute silencing of the dopaminergic neurons caused an increase in walking speed: flies crossed the midline more often (Fig. 2, Ai), but the time spent moving in the chamber remained unchanged (Fig 2, Bi) when compared to temperature effects observed in the genetic control group. Another dramatic effect of manipulating DA neuron function was seen in courtship behavior, where experimental males at the elevated temperature appeared very uninterested in courting females (see Movies S2, S3). Their courtship index was greatly reduced (Fig 2, Ci), even though the experimental males at the elevated temperature continuously moved around the courtship chamber.

### Genetic background and the presence of the *white^1118^* mutation do not account for the aggression phenotype

To match genetic backgrounds of the *Gal4* drivers utilized in these studies, we used either *w^1118^* or *w^1118^(CS)* males in carrying out the genetic control crosses with *UAS-sh^ts1^*. A number of laboratories have reported that changes in *white* (*w*) function (codes for an ABC transporter protein) can have effects on male-male courtship [34]–[37], male aggression [38], learning [39] and memory [26], and also can cause changes in the levels of monoamines in fly heads [26], [40]. To deal with possible variability in our results due to the *white* gene, we designed genetic controls that had the same *w^1118^* mutation and the same numbers of mini-w transgenes, differing therefore only in their genetic backgrounds. To our surprise, we found that pairs of *w^1118^(CS);Shi^ts1^/+* flies established fewer dominance relationships than *w^1118^; Shi^ts1^/+* pairs at both 19°C and 30°C (Table 1). To ask whether the genetic background change might cause differences of this type in baseline dominance rates, we measured monoamine levels and aggressive behavior in flies with: 1) both *w^1118^* and white genetic background (*w^1118^; Shi^ts1^/+*), 2); *w^1118^* and CS genetic background (*w^1118^(CS);Shi^ts1^/+*); and 3) *w^+^* and CS genetic background (*CS; Shi^ts1^/+*). No differences were observed in the rates of lunging and the establishment of dominance relationships in these 3 groups of flies when tested at 25°C (Table S1). Furthermore, all groups had similar levels of DA and 5HT in brains, as measured by HPLC (data not shown). Other investigators also have reported no differences in DA and 5HT levels between *w^1118^* and *w^+^* flies [41], [42]. Thus, neither the presence of a *w^1118^* mutation, nor the genetic background, seemed to influence aggressive behavior at the normal 25°C incubation conditions.

## Discussion

In this report, we utilized *Drosophila* as a model system to explore separately the roles of 5HT and DA in aggression in flies. For this purpose, we generated new *TRH-Gal4* driver lines to direct expression of effector genes selectively in 5HT-containing neurons. The behavioral consequences of impaired serotonergic neuron function were compared and contrasted with similar targeted disruption of dopaminergic neurotransmission using a *TH-Gal4* driver, or by interference with neurotransmission of both amines using a *DDC-Gal4* driver. We used these lines to drive expression of the dominant negative temperature sensitive Dynamin effector gene (using *UAS-shi^ts1^*) because it allowed potent, at least partially reversible, *acute* alteration of synaptic transmission in adult flies [43]. Via this route we could avoid possible developmental complications resulting from *chronic* changes in monoaminergic function.

### Combined and separate roles of dopamine and serotonin in fruit fly social behavior

Simultaneous disruption of DA and 5HT neurotransmission using the *DDC-Gal4* driver resulted in flies that continuously engaged in short low-intensity interactions. Even though we observed almost a three-fold increase in the numbers of encounters between the flies, escalation to higher-intensity levels of aggression never was seen: these flies did not lunge or establish dominance. Selective disruption of either 5HT or DA neurotransmission alone failed to duplicate this phenotype. With the new *TRH-Gal4* line, we demonstrated that serotonergic neuron function is involved in the escalation of fights to higher intensity levels. First, we showed that flies with reduced serotonergic neurotransmission had normal levels of overall activity and close to normal numbers of encounters, but displayed substantial reductions in male mid-intensity aggression, including “lunging” and “holding”. As a result, fewer fights ended up in dominance relationships. Next, we demonstrated that flies with activated serotonergic cells progressed to mid-intensity aggression faster, exhibiting high numbers of lunges throughout fights, including after the establishment of dominance. This finding fits in with ongoing theory that normal adaptive forms of aggression are positively related to 5HT neuronal activity [44]. Selective interference with dopaminergic neurotransmission produced flies that did not land on the food cup in our standard aggression assay. In smaller fighting chambers, these flies successfully landed on the food cup, but still rarely engaged in any kind of social interaction. Disruption of dopaminergic neurotransmission also produced decreased courtship and increased locomotion. Constitutive inhibition of dopaminergic neurotransmission using tetanus toxin [22] yielded adult flies that were hyper-excited upon being startled, and that required an abnormally long time to calm down. The baseline locomotion of these flies was normal, possibly due to compensatory mechanisms occurring during development. In our experiments, in which we interfered with DA neuron function acutely, transferring flies to the arena alone might produce hyper-excitability. In a hyper-excited state, male flies might ignore females in courtship chambers or other males in fighting chambers. These results support the suggestion that dopaminergic neurons normally might inhibit circuitry concerned with behavioral excitability in the *Drosophila* nervous system. This does not explain, however, why blocking synaptic transmission in both DA and 5HT neurons using the *DDC-Gal4* driver, does not duplicate the phenotype. One possible caveat is that the *DDC-Gal4* line can drive expression in small numbers of neurons that do not appear to contain DA or 5HT [26], [30]. Further study could address the role of these neurons in mediating aggressive behavior. Another possibility is that 5HT and DA have opposite actions on certain of the shared targets, and changing the function of both amines simultaneously yields a phenotype different from the one obtained by manipulating each of the amines alone. To explore this further, we analyzed the aggressive behavior of flies that had the *UAS-Shi^ts1^* transgene expressed under combined control of both *TH-GAL4* and *TRH-GAL4*. We found that the *TRH-Gal4/+;TH-Gal4/UAS-Shi^ts1^* males at the restrictive temperature behaved similarly to *TH-Gal4/UAS-Shi^ts1^* flies, constantly moving around in the fighting chamber, rarely landing on the food cup or engaging in social interactions (preliminary data). This finding suggests that the dopaminergic “hyperactivity” phenotype may override the serotonergic phenotype when neurotransmission is disrupted in this way in both amine systems. However, any interpretation should take into account the fact that the effectiveness of various GAL4 drivers may differ depending on their target sites and the transgene expression levels. Further speculation on differences in phenotypes observed when using the *DDC-*, *TH-* or *TRH-GAL4* drivers in various combinations may be unwarranted with the information currently available.

### The dynamic aspects of fights

The experimental arena used in our aggression assays [1] was designed to allow flies to compete for resources within a space that allows a losing fly to retreat. In such an arena we can ask how long it takes to initiate fights, what intensity levels are reached, and whether a dominance relationship is established as a consequence of a fight. Fly fights involve a series of brief encounters during which animals interact and separate repeatedly within a specified time window. Socially naive wild-type male flies begin interactions by displaying low-intensity behavioral patterns like frequent approach and fencing that contributes to the higher numbers of encounters observed within the initial 20 min of a fight (Yurkovic et al, 2006; Fig. 1A). As fights escalate, both combatants are capable of displaying mid- and high-intensity behavioral patterns, like lunge, hold, boxing and tussling. After dominance is established, only winners lunge. Defeated flies no longer lunge and continually retreat from the food surface when confronted by winners [3]. By ignoring the dynamics of fights, and asking only whether a pair of flies show lunging behavior, much information is lost.

To illustrate, Dierick and Greenspan [25] showed that by raising 5HT levels in flies either by feeding 5-hydroxytryptophan or by constitutive over-expression of dTRH in DDC-positive neurons a higher percentage of fly pairs showed enhanced aggression. Whether such increase was present continuously throughout fights, or whether hierarchical relationships were established during these fights was not considered. Since lunging behavior changes during fights depending on the outcome and the response of opponent flies, it is difficult to interpret results showing increases in aggressive behavior in a higher percentage of fights. Moreover, reductions in 5HT neuron function by feeding a biosynthetic inhibitor (α-methyl tryptophan), or by silencing DDC-positive neurons via tetanus toxin expression had no effects on fighting frequency in their experiments. Studies in other invertebrate systems (crayfish, lobsters) have used acute and chronic pharmacological treatments to change amine levels [45], [46]. Those results are difficult to interpret and compare with the present data, as elevations and reductions of 5HT levels can have different effects under different experimental treatment regimes. Our current results demonstrate clearly that acute reduction of serotonergic transmission and acute activation of serotonergic neurons yield opposite behavioral phenotypes, changing fly's ability to escalate fights.

### Why use acute interference with aminergic neurotransmission?

In these studies we chose to induce acute rather than constitutive changes in neurotransmission. Amines like 5-HT appear early in development of the nervous system in many species of animals, and have been shown to serve a variety of developmental roles [47]–[52]. By altering monoaminergic transmission throughout development, any variety of changes in dopaminergic or serotonergic neuron function might be seen later in development or in adulthood. To illustrate, Chang et al [53] showed that chronic over-expression of the vesicular monoamine transporter in DDC-positive neurons, which should cause an increased loading of amines into vesicles, ultimately led to a reduced response to cocaine administration in adults. The authors suggested that this might be caused by compensatory changes in the sensitivity of amine receptors or in the efficiency of amine uptake in adults, that resulted from increased amine release during embryonic or larval life.

### Advantages of the use of a TRH-Gal4 line to examine serotonergic neuron function

We sought to generate a selective, well characterized *TRH-Gal4* line in order to address serotonergic function, rather than combining the *DDC-* and *TH-Gal4* drivers and subtracting results from each other. One complication of the latter approach is that serotonergic and dopaminergic systems may assume each other's function under certain experimental or natural conditions. In *Drosophila*, for example, ectopic expression of the serotonin transporter molecule (dSERT) leads to the ability to store 5HT in serotonergic and in dopaminergic neurons in the nerve cord [54]. This suggests that the machinery for transmitter storage is similar in 5HT- and DA-containing neurons, and different from that found in other types of neurons where uptake of amines is not observed. Other examples in the literature suggest that, under certain experimental conditions, serotonergic neurons can release DA. In the progressive dopaminergic neuron degeneration seen in Parkinson's disease, for example, fewer dopaminergic nerve terminals are available to take up and metabolize the L-DOPA used to treat early stages of the disease. It has been suggested that under these circumstances, serotonergic neurons might store and release DA in addition to 5HT [55]–[57] It is known that the aromatic L-amino acid decarboxylase enzyme in serotonergic neurons can convert L-DOPA to DA [58] and that dopaminergic and serotonergic neurons contain a single type of vesicular monoamine transporter (VMAT-2, [59]). Recent experiments suggest that unregulated DA release from serotonergic nerve terminals might be a prime cause of dyskinesia seen in a rat model of Parkinson's disease [60].

Using the new *TRH-GAL4* line we were able to selectively visualize the 5-HT circuitry in the CNS of adult *Drosophila*. This allowed us to begin to speculate on which of the serotonergic neurons or the sub-circuits could be involved in controlling aggression. Several previous reports centered on the localization, arborization and/or identity of small numbers of individual setotonergic neurons in the *Drosophila* CNS. To illustrate, two clusters of cells in abdominal ganglion were immunopositive both for 5-HT and the male forms of Fruitless (Fru^M^) [61]–[63]. These clusters send projections that innervate parts of the male reproductive apparatus concerned with seminal fluid and sperm transfer. We co-stained *TRH-Gal4/UAS-mCD8∶GFP* brains with an anti-FRU^M^ antibody and found no additional Fru^M^ positive 5HT cells. In the central brain, several large pairs of 5HT neurons are found in the SOG (from the SE1 and SE2 clusters). These cells resemble a recently identified pair of motor neurons [64] that are activated by sugar and inhibited by bitter compounds, and that have been reported to be necessary and sufficient for a specific subprogram of the proboscis extension reflex. In the same SE1 cluster from SOG, two large 5HT neurons send heavy descending projections into the ventral nerve cord that resemble the projection patterns of 5HT-containing neurons identified in the blowfly [65]. Those neurons projecting to the dorsal surface of the thoracicoabdominal ganglia have been suggested to release 5HT into the circulation. In preliminary experiments, we observe that genetic ablation of what appear to be the same 5HT-neurons, yields flies that have difficulties in proboscis extension behavior during feeding. The ability of these flies to lunge and display aggression, however, remains intact. A reasonable assumption would be that 5HT-positive cells sending arbors of endings to the central complex and the mushroom bodies might be involved in the regulation of aggression. In this regard, two of the known types of serotonin receptors in *Drosophila*, 5-HT1B [66] and 5-HT1A [24], appear to be highly expressed in mushroom bodies, suggesting important roles for serotonergic neurons in mushroom body function. At present, we are carrying out experiments that ask whether any of the behavioral effects described in this paper are mediated by 5HT1B or 5HT1A receptors.

In summary, by generating genetic tools that allowed us to selectively manipulate serotonergic neuron function without changing dopaminergic neurons, we collected evidence for a specific role of 5HT in aggressive behavior in fruit flies. Serotonin is not required to initiate fights, but is necessary for the proper escalation of aggression to higher intensity levels and thereby to facilitate the establishment of hierarchical relationships. Further studies will be required to identify the subsets of 5HT neurons involved in these processes and to begin to unravel the circuitry involved.

## Methods

### Fly Stocks and crosses

The following fly lines were used in this study: *DDC-Gal4* from J. Hirsh (University of Virginia, Charlottesville, VA), *TH-Gal4* from Serge Birman (Developmental Biology Institute of Marseille, Marseille, France), *UAS-dTrpA1* from Paul Garrity, *UAS-shi^ts1^*, *w^1118^*, Canton-S, *UAS-nls∶GFP* and *UAS-mCD8∶GFP* from the Bloomington Stock Center (Bloomington, IN). The *TRH-Gal4* line was generated as described below. To obtain experimental flies *DDC-Gal4*, *TH-Gal4* and *TRH-Gal4* males were crossed to *UAS-shi^ts1^* or *UAS-dTrpA1* females. *w^1118^* males crossed to *UAS-shi^ts1^* females were used as genetic background controls for the *DDC-Gal4* and *TH-Gal4* experiments. In “genetic control” crosses for the *TRH-Gal4* experiments, *w^1118^* males were replaced by *w^1118^(CS)* males in order to match the genetic background of the *TRH-Gal4* flies (see below).

### *TRH-Gal4* constructs

A collection of *TRH-Gal4* lines containing either long or short regulatory sequences from the Trh gene (CG9122) was generated using a P-element strategy. BAC DNA 98-17O7 was used as a template for PCR with Phusion DNA polymerase (NEB, Ipswich, MA) and 5′-phosphorylated primers: SM(1A)/BI(1S) was used for the long and SM(1A)/SM(1S) for the short fragment. The long 1.7 kb and short 0.7 kb PCR products were ligated into NotI-digested, blunted and dephosphorylated pMB3 vectors upstream of the Gal4 cassette.

Primers: SM(1A)-CTTGGTAGCTACTCGTTTTCGATTTCCGC; BI(1S)-ATAAAAGT-AAATATCTGGTACGACATTTG; SM(1S)-CCAGCCTGACCACCCGGCCCACCCAACG.

The final construct DNAs were injected into *y^−^w^−^* embryos (using the Cutaneous Biology Research Center (CBRC) Transgenic *Drosophila* Core, MGH, Boston, MA). These yielded 10 *TRH*-*short* and 2 *TRH-long Gal4* viable and fertile lines. To eliminate any behavioral effects caused by the *y^−^w^−^* background, flies were backcrossed to *w^1118^(CS)* line for 6 generations using the scheme described earlier [67].

### Immunohistochemistry

Adult male brains were dissected, fixed, treated with primary and secondary antibodies, and prepared for confocal imaging as described previously [68]. The following primary antibodies were used: mouse anti-GFP (1∶1000) (Invitrogen, Carlsbad, CA), rabbit anti-5-HT (1∶1000) (Sigma-Aldrich, St. Louis, Missouri) and rabbit anti-TH (1∶250) (Novus Biologicals, Littleton, CO). The secondary antibodies used included: Alexa Fluor 488- and Alexa Fluor 594-conjugated cross-adsorbed antibodies (Invitrogen, Carlsbad, CA). Confocal Z-stacks were acquired using an Olympus Fluoview FV1000 confocal microscope with a UAPO 20x water-immersion objective, and processed with ImageJ software.

### Behavioral Assays

Flies were reared on a standard cornmeal medium at 19°C and 50% relative humidity on a 12∶12 hr light∶dark cycle. Pupae were picked and placed in isolation in individual 16×100 mm glass vials containing 1.5 ml of standard food medium, where they were allowed to emerge as adults and kept for 5–7 days before testing. One day before the aggression assays, flies were anesthetized with CO_2_, a small dot of acrylic paint was placed on the thorax, and the flies were returned to their isolation vials to recover. For *UAS-shi^ts1^* experiments, one set of flies was tested at 19°C, the temperature at which the flies had been reared; a second set was transferred in their isolation vials to a 30°C experimental room one hour before the beginning of the test. Earlier studies in our laboratory had shown that this was an adequate time to observe effects triggered by the high temperature treatment. For UAS-dTrpA1 data, both genetic control and experimental flies were transferred to a 26°C experimental room 15 min before the test. A low-temperature control was omitted in this set of experiments because a 26°C temperature is considered normal for flies and does not lead to the kinds of behavioral changes seen at 30°C. All experiments were performed within the first 2.5 hr after lights-on.

#### Aggression assays

Two males of the same genotype and the same age were paired and allowed to interact for 60 min in an experimental fight arena as described previously [1]. The behaviors scored for pairs of animals were: numbers of encounters, total duration of encounters and the average time of a single encounter calculated from the first two parameters. The individual behavioral patterns scored for each fly included: low-intensity behavioral components (approach, front and side fencing, wing threat, retreat and pursuit); mid-intensity components (lunge, hold, chase, charge); and non-aggressive components (preening and male-male courtship). Higher-intensity patterns like boxing and tussling were not seen in the flies used in these studies at either the permissive or restrictive temperatures. The one hr of data collected was divided into three 20 min time bins to examine the dynamics of fights, which are known to change over the course of a fight [3].

#### Courtship assays

Courtship assays were performed in round chambers (10 mm in diameter, 5 mm in height). A single experimental male and a virgin CS female were placed in the chamber and observed for 10 min. The Courtship Index (CI) was calculated as the fraction of time that a male spent courting the female (includes tapping, wing extension and vibration, attempted copulation) during the observation period.

#### Locomotion assays

Locomotion was measured by placing single flies into a 10 mm diameter chamber with a bisecting line on the bottom. The total number of midline crosses and the total time spent moving within a 5 min observation period were scored as a measure of locomotor activity [69].

#### Geotaxis and phototaxis assays

Both assays were performed as described earlier [70] using a slightly modified version of a maze that incorporates five consecutive choices [71]. Geotaxis and phototaxis scores were measured separately for each fly.

### Statistical Analyses

All data were analyzed using the SPSS 16.0 for Mac statistical software package (SPSS, Chicago, IL). For pairwise comparisons the nonparametric two-independent-sample Mann-Whitney test was used. Two-tailed P values were determined with the significance level set at P<0.05. Non-significant trends were reported for P<0.1.

## Supporting Information

Figure S1 Expression patterns of other TRH-Gal4 lines. (A–C) Co-localization of 5HT immunostaining (red) and nuclear nls∶GFP (green) driven by TRH-Gal4 line 2 (on 2nd chromosome, derived from the long regulatory sequence of the TRH gene) in an adult male brain. (D–E) Examples of a partial overlap of 5HT immunostaining (red) and nuclear nls∶GFP (green) driven by TRH-Gal4 lines derived from the short regulatory sequence of the Trh gene (see Methods). The white arrows point to 5HT-positive cells not labeled by GFP in different lines: PMP clusters in (D); SE1, SE2 and PMP clusters in (E); AMP and PMP clusters in (F); PMP clusters in (G); PMP and SE2 clusters in (H); SE2, PMP and AMP clusters in (I).
(6.48 MB TIF)

Figure S2 Close-up view of 5HT clusters visualized by two serotonin-specific GAL4 lines. (A) TRH-Gal4 driven expression of nuclear nls∶GFP, (B) TPH- Gal4 [23] driven expression of nuclear nls∶GFP in Drosophila brain. 5HT immunostaining is shown in red, anti-GFP staining is shown in green. White arrows point to individual 5HT cells not labeled by GFP. Only clusters with the most obvious differences between the two GAL4 lines are shown (for quantifications, see Table S2).
(5.65 MB TIF)

Table S1 The w1118 genetic background does not affect aggressive behavior. w1118, w1118(CS) and CS males were crossed to UAS-shi(ts1) females to examine the influence of the w1118 genetic background on aggressive behavior. Socially-naïve progeny males of each genotype were paired and allowed to interact for 60 min in our standard fighting chambers at 25°C. Same genotype pairings all landed on the food cup, lunged and established dominance normally.
(0.06 MB DOC)

Table S2 Numbers of neurons labeled by different genetic tools in various 5HT clusters in fly brains. TRH-Gal4 and TPH-Gal4 lines were crosses to UAS-nls∶GFP, brains of progeny males were dissected, stained and imaged using confocal microscope as described in Methods. Data are presented as Mean ± SEM per hemisphere. Percentage of 5HT positive cells labeled by each genetic approach is shown in parentheses for each cluster. Clusters with most apparent differences are highlighted in gray color. * - Individual neurons in SE2 and SE3 clusters in some brains were difficult to discern which led to lower cell counts in SE2 and higher cell counts in SE3. DDC-Gal4; TH-GAL80 data were taken from [26] for comparison purposes only. These data were obtained using females, no statistics were presented in the original paper.
(0.10 MB DOC)

Movie S1 Motor dysfunction phenotype in flies with disrupted serotonergic neurotransmission.
(12.29 MB MOV)

Movie S2 Normal courtship in genetic control males (*UAS-Shi^ts1^/+, 30°C*).
(5.11 MB MOV)

Movie S3 Decreased courtship in males with disrupted dopaminergic neurotransmission (*TH-Gal4/UAS- shi^ts1^, 30°C*).
(3.73 MB MOV)
