# Supplementary figures and images for: Targeted Manipulation of Serotonergic Neurotransmission Affects the Escalation of Aggression in Adult Male Drosophila melanogaster
Source: PLoS One. 2010 May 24;5(5):e10806. doi: 10.1371/journal.pone.0010806 (PMC2875409; doi:10.1371/journal.pone.0010806)

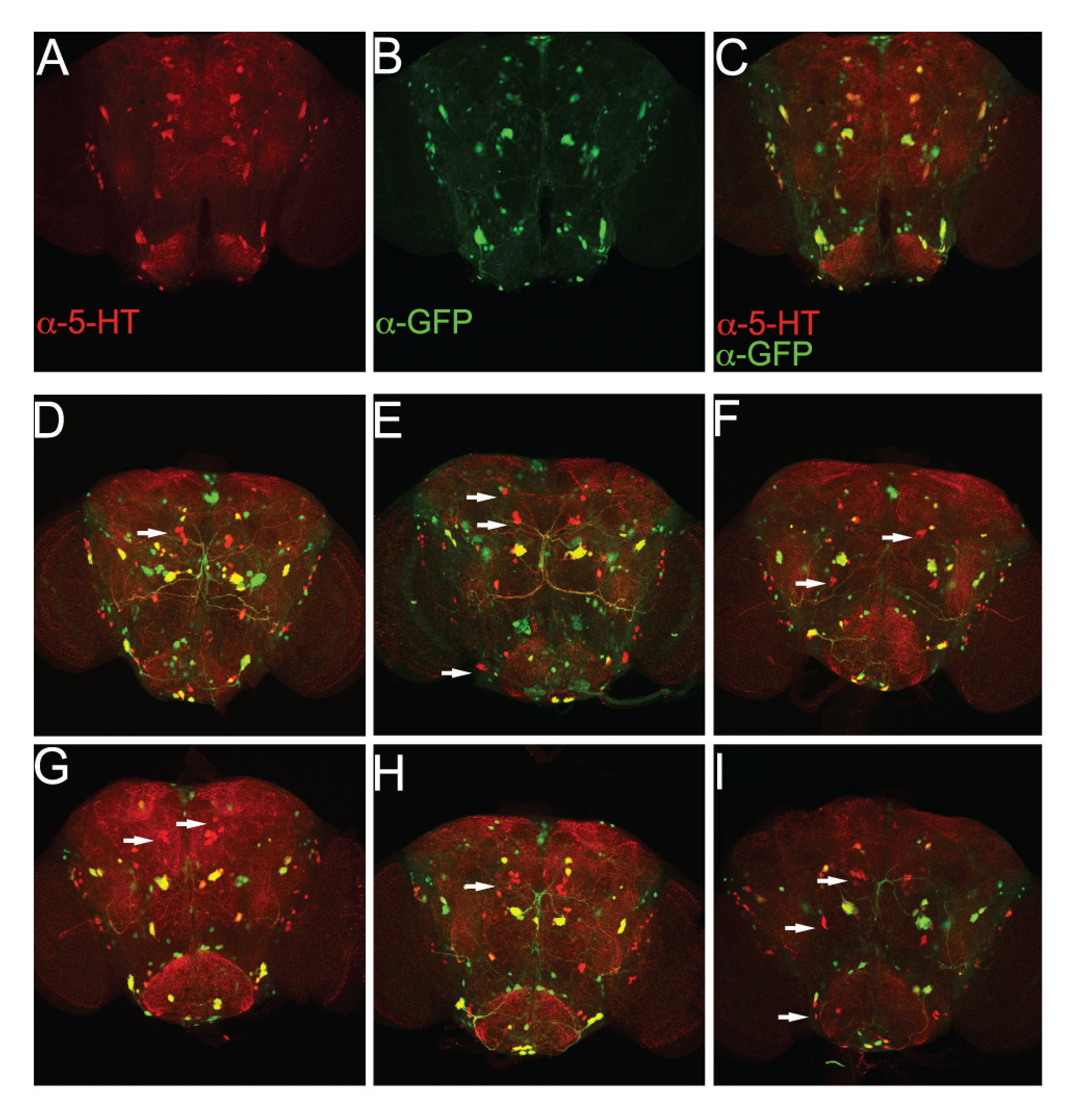

Supplement: Figure S1 — Expression patterns of other TRH-Gal4 lines. (A–C) Co-localization of 5HT immunostaining (red) and nuclear nls∶GFP (green) driven by TRH-Gal4 line 2 (on 2nd chromosome, derived from the long regulatory sequence of the TRH gene) in an adult male brain. (D–E) Examples of a partial overlap of 5HT immunostaining (red) and nuclear nls∶GFP (green) driven by TRH-Gal4 lines derived from the short regulatory sequence of the Trh gene (see Methods). The white arrows point to 5HT-positive cells not labeled by GFP in different lines: PMP clusters in (D); SE1, SE2 and PMP clusters in (E); AMP and PMP clusters in (F); PMP clusters in (G); PMP and SE2 clusters in (H); SE2, PMP and AMP clusters in (I). (6.48 MB TIF) [file pone.0010806.s001.tif]

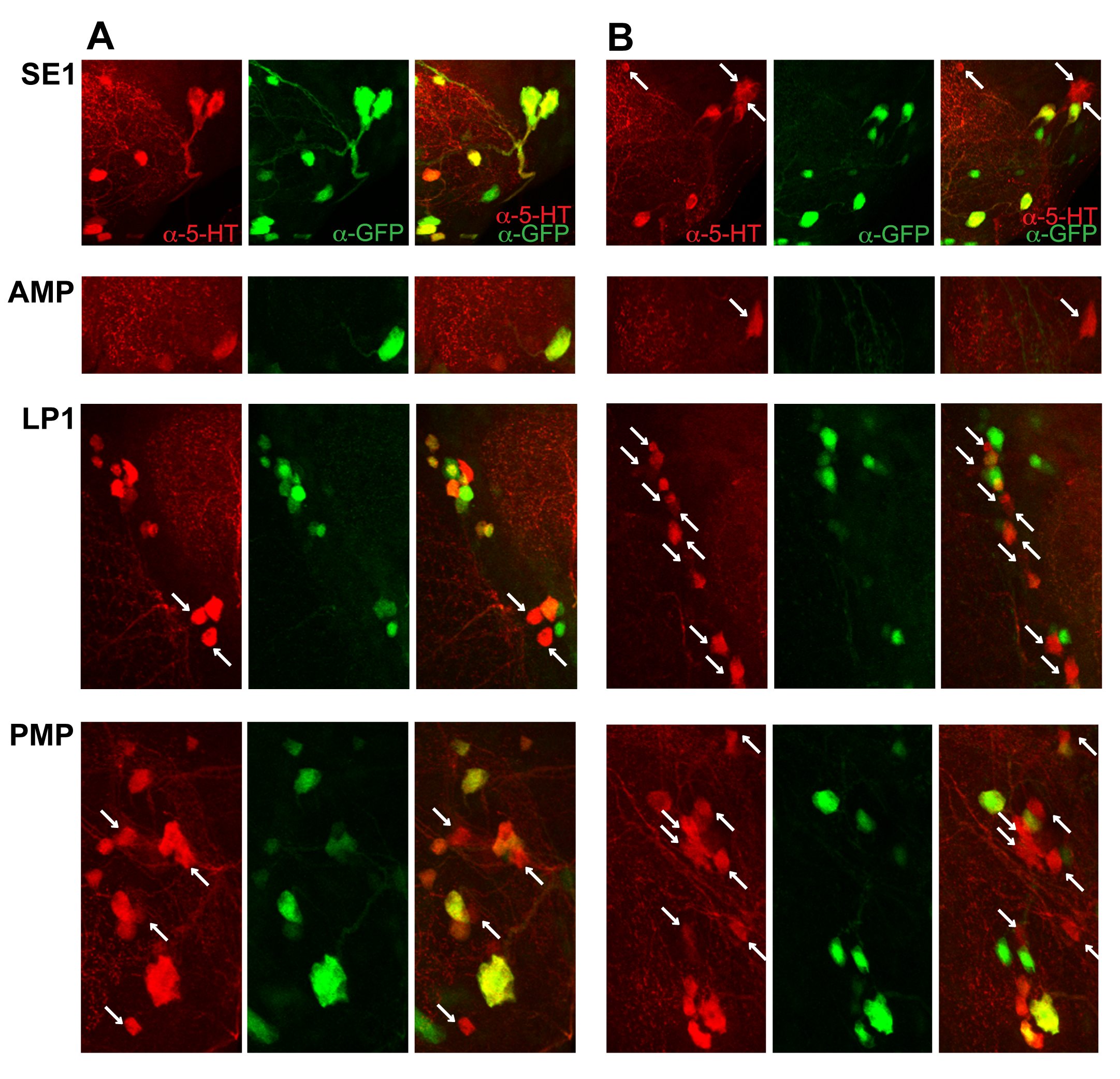

Supplement: Figure S2 — Close-up view of 5HT clusters visualized by two serotonin-specific GAL4 lines. (A) TRH-Gal4 driven expression of nuclear nls∶GFP, (B) TPH- Gal4 [23] driven expression of nuclear nls∶GFP in Drosophila brain. 5HT immunostaining is shown in red, anti-GFP staining is shown in green. White arrows point to individual 5HT cells not labeled by GFP. Only clusters with the most obvious differences between the two GAL4 lines are shown (for quantifications, see Table S2). (5.65 MB TIF) [file pone.0010806.s002.tif]
