# Supplementary material for: Targeted Manipulation of Serotonergic Neurotransmission Affects the Escalation of Aggression in Adult Male Drosophila melanogaster
Source: PLoS One. 2010 May 24;5(5):e10806. doi: 10.1371/journal.pone.0010806 (PMC2875409; doi:10.1371/journal.pone.0010806)
Supplement: Table S1 — The w1118 genetic background does not affect aggressive behavior. w1118, w1118(CS) and CS males were crossed to UAS-shi(ts1) females to examine the influence of the w1118 genetic background on aggressive behavior. Socially-naïve progeny males of each genotype were paired and allowed to interact for 60 min in our standard fighting chambers at 25°C. Same genotype pairings all landed on the food cup, lunged and established dominance normally. (0.06 MB DOC) [file pone.0010806.s003.docx]

**Table S1.** **The w*^1118^* genetic background does not affect aggressive behavior.**

|  | ***w^1118^;Shi^ts1^/+*** | ***w^1118^(CS);Shi^ts1^/+*** | ***CS;Shi^ts1^/+*** |
| --- | --- | --- | --- |
| ***Number of fights*** | n=10 | n=10 | n=10 |
| ***Land on surface*** | 100% | 100% | 100% |
| ***Lunge during fight*** | 100% | 100% | 100% |
| ***Establish dominance*** | 90% | 80% | 80% |

*w^1118^,* *w^1118^(CS)* and *CS* males were crossed to *UAS-shi^ts1^* females to examine the influence of the *w^1118^* genetic background on aggressive behavior. Socially-naïve progeny males of each genotype were paired and allowed to interact for 60 min in our standard fighting chambers at 25°C. Same genotype pairings all landed on the food cup, lunged and established dominance normally.
