# Supplementary material for: Targeted Manipulation of Serotonergic Neurotransmission Affects the Escalation of Aggression in Adult Male Drosophila melanogaster
Source: PLoS One. 2010 May 24;5(5):e10806. doi: 10.1371/journal.pone.0010806 (PMC2875409; doi:10.1371/journal.pone.0010806)
Supplement: Table S2 — Numbers of neurons labeled by different genetic tools in various 5HT clusters in fly brains. TRH-Gal4 and TPH-Gal4 lines were crosses to UAS-nls∶GFP, brains of progeny males were dissected, stained and imaged using confocal microscope as described in Methods. Data are presented as Mean ± SEM per hemisphere. Percentage of 5HT positive cells labeled by each genetic approach is shown in parentheses for each cluster. Clusters with most apparent differences are highlighted in gray color. * - Individual neurons in SE2 and SE3 clusters in some brains were difficult to discern which led to lower cell counts in SE2 and higher cell counts in SE3. DDC-Gal4; TH-GAL80 data were taken from [26] for comparison purposes only. These data were obtained using females, no statistics were presented in the original paper. (0.10 MB DOC) [file pone.0010806.s004.docx]

**Table S2.** **Numbers of neurons labeled by different genetic tools in various 5HT clusters in fly brains.**

|  | ***TRH-Gal4 on 3^rd^ (n=10)*** | | ***TPH-Gal4 (n=6)*** | | ***DDC-Gal4; TH-GAL80*** | |
| --- | --- | --- | --- | --- | --- | --- |
|  | *current work* | | *line from [23]* | | *adopted from [26]* | |
| ***Clusters*** | ***5HT*** | ***5HT+ TRH-GAL4*** | ***5HT*** | ***5HT+ TPH-GAL4*** | ***5HT*** | ***5HT + DDC-GAL4*** |
| **SE1** | **2.8±0.4** | **2.8±0.4 (100%)** | **4.8±0.5** | **2.8±0.5 (59%)** | **3** | **2 (67%)** |
| SE2 * | 0.7±0.3 | 0.7±0.3 (100%) | 0.7±0.4 | 0.7±0.4 (100%) | 3 | 3 (100%) |
| SE3 | 6.2±0.7 | 6.0±0.6 (97%) | 8.7±1.9 | 7.3±1.2 (85%) | 3 | 3 (100%) |
| **AMP** | **1.0±0.0** | **1.0±0.3 (100%)** | **1.0±0.0** | **0.0±0.0 (0%)** | **1** | **1 (100%)** |
| ALP | 2.4±0.3 | 2.1±0.3 (88%) | 2.7±0.2 | 2.0±0.4 (75%) | 3 | 3 (100%) |
| **LP2** | **7.5±1.0** | **6.6±0.8 (88%)** | **10.7±1.2** | **5.0±0.9 (47%)** | **9-11** | **3-4 (33-36%)** |
| PLP | 1.8±0.1 | 1.7±0.2 (94%) | 1.5±0.3 | 1.3±0.3 (89%) | 3 | 3 (100%) |
| **PMP** | **12.5±1.1** | **9.4±0.7 (75%)** | **15.7±0.5** | **8.8±0.3 (56%)** | **13-14** | **8-10 (61-71%)** |

*TRH-Gal4* and *TPH-Gal4* lines were crosses to *UAS-nls:GFP*, brains of progeny males were dissected, stained and imaged using confocal microscope as described in Methods. Data are presented as Mean ± SEM per hemisphere. Percentage of 5HT positive cells labeled by each genetic approach is shown in parentheses for each cluster. Clusters with most apparent differences are highlighted in bold. * - Individual neurons in SE2 and SE3 clusters in some brains were difficult to discern which led to lower cell counts in SE2 and higher cell counts in SE3. *DDC-Gal4; TH-GAL80* data were taken from [26] for comparison purposes only. These data were obtained using females, no statistics were presented in the original paper.
